# Development and validation of IIKC: an interactive identification key for *Culicoides* (Diptera: Ceratopogonidae) females from the Western Palaearctic region

**DOI:** 10.1186/1756-3305-5-137

**Published:** 2012-07-09

**Authors:** Bruno Mathieu, Catherine Cêtre-Sossah, Claire Garros, David Chavernac, Thomas Balenghien, Simon Carpenter, Marie-Laure Setier-Rio, Régine Vignes-Lebbe, Visotheary Ung, Ermanno Candolfi, Jean-Claude Delécolle

**Affiliations:** 1 IPPTS, Université de Strasbourg, EA 4438, 67000, Strasbourg, France; 2 Cirad, UMR Contrôle des maladies, 34398, Montpellier, France; 3 Institute for Animal Health, Pirbright, GU24 0NF, United Kingdom; 4 EID-Méditerranée, 34184, Montpellier, France; 5 UMR 7207 CNRS-MNHN-Université Pierre et Marie Curie, 75005, Paris, France

**Keywords:** Multi-entry key, Identification key, Interactive key, Bluetongue, African horse sickness, Culicoides, Vectors

## Abstract

**Background and methods:**

The appearance of bluetongue virus (BTV) in 2006 within northern Europe exposed a lack of expertise and resources available across this region to enable the accurate morphological identification of species of *Culicoides* Latreille biting midges, some of which are the major vectors of this pathogen. This work aims to organise extant *Culicoides* taxonomic knowledge into a database and to produce an interactive identification key for females of *Culicoides* in the Western Palaearctic (IIKC: Interactive identification key for *Culicoides*). We then validated IIKC using a trial carried out by six entomologists based in this region with variable degrees of experience in identifying *Culicoides*.

**Results:**

The current version of the key includes 98 *Culicoides* species with 10 morphological variants, 61 descriptors and 837 pictures and schemes. Validation was carried out by six entomologists as a blind trial with two users allocated to three classes of expertise (beginner, intermediate and advanced). Slides were identified using a median of seven steps and seven minutes and user confidence in the identification varied from 60% for failed identifications to a maximum of 80% for successful ones. By user class, the beginner group successfully identified 44.6% of slides, the intermediate 56.8% and the advanced 74.3%.

**Conclusions:**

Structured as a multi-entry key, IIKC is a powerful database for the morphological identification of female *Culicoides* from the Western Palaearctic region. First developed for use as an interactive identification key, it was revealed to be a powerful back-up tool for training new taxonomists and to maintain expertise level. The development of tools for arthropod involvement in pathogen transmission will allow clearer insights into the ecology and dynamics of *Culicoides* and in turn assist in understanding arbovirus epidemiology.

## Background

During the last decade, the decline of fundamental entomological taxonomic expertise has become an increasing concern worldwide and has impacted directly upon disciplines as diverse as biodiversity conservation [1], medical and veterinary entomology [2,3] and pest management [4]. The correct classification of subject species is a vital prerequisite to any biological study and is a primary requirement for comparability across studies. Despite this, morphological taxonomy, which is by far the most commonly used means of identification used by biologists worldwide, receives relatively little financial support.

Ideally, identification of a biological specimen can be conducted using direct comparison with existing named specimens, including the original type. This comparative approach is feasible only when type locality is known accurately, the original specimen has been suitably preserved, and species description written in easy-access articles. While this is possible in larger institutions with a long track record of experimentation on a specific taxon, it is more common for the specimen to be compared to written descriptions and whatever identifying material (e.g. photographs, diagrams etc), is available through previously published work. The power of identification of groups of related organisms through the use of contrasting statements concerning morphological characters, also known as identification keys, was first realised by Lamarck (1778).

The development of electronic communications has revolutionised taxonomy worldwide, initially through facilitating contact between workers worldwide and more recently by allowing the open-access publication of taxonomic data. In addition, a large number of interactive keys allowing accurate identification of vector species and groups are increasingly available, either by downloading or directly through websites (e.g. Phlebotomine sandflies key [5] tsetse flies [6] and mosquitoes [7,8]). These not only allow direct sharing of information, but also provide a powerful training tool where specialised expertise is otherwise reliant upon single individuals.

The recent unprecedented bluetongue virus (BTV) outbreaks in Western Europe [9] illustrate how a relatively neglected arthropod vector group can rapidly increase in interest. BTV causes bluetongue (BT), a disease that affects wild and domestic ruminants, and the virus is biologically transmitted by various species of *Culicoides* Latreille biting midges (Diptera: Ceratopogonidae). At the time of introduction of BTV in 2006 to much of Western Europe, the number of groups working on *Culicoides* in Europe was small. Following the incursion, there was a substantial need to rapidly train workers in *Culicoides* taxonomy and this was in part accomplished through the use of online resources (e.g. http://www.culicoides.net) and direct training by the limited number of experts available. It was clear, however, that improvements could be made to this system from the following observations: (1) many workers had difficulty identifying the diversity of *Culicoides* present in their samples (particularly those species that did not fall within what were perceived to be the main vector groups), (2) many lacked either appropriate identification tools, or did not know where to find them (3) there was a lack of continuity and successive planning in preserving skills in taxonomy within countries, preventing the building of local expertise in *Culicoides* taxonomy [10].

In the case of the Western Palaearctic biting midge fauna, Campbell & Pelham-Clinton [11] and Kremer [12] (in French) contain the only dichotomous keys covering a wide range of species. In addition, Delécolle [13] (in French) published a revised version of Kremer [12] for a limited number of species from the northeast of France. These keys covering only restricted geographical areas, do not contain the most recent synonyms or the new species records, are entirely dichotomous, and therefore are limited in terms of use for non specialists. The aim of this work therefore, is to organise extant taxonomic knowledge for the Western Palaearctic fauna into a database in order to create the first Interactive Identification Key (IIKC) for *Culicoides* females. Initially started in the framework of the European project MedReoNet [14], this key was tested using a ring trial with 37 specimens being sent to six users from three different institutes and with different levels of expertise, with the objective of defining the descriptors required for accurate identification and evaluating the importance and efficiency of the key. The freely shared e-taxonomy knowledge is discussed as a powerful tool to fill in the current taxonomic impediment to progress in understanding *Culicoides* ecology and hence arbovirus epidemiology.

## Methods

### Biological material, illustrations and choice of descriptors

Taxonomic information was collated from 98 slide-mounted *Culicoides* ( Additional file 1). Twenty of these species were characterised from types preserved in the Callot and Kremer collection (Strasbourg, France). Data on *C. paradisionensis* was obtained from the type specimen in the Delécolle collection (Strasbourg, France), whereas the 77 other species were studied from specimens kept in the collection at IPPTS (Strasbourg, France). To ensure the reliability of the key, uncertainty due to intraspecific variation in morphology was avoided by coding some descriptors as polymorphic to ensure users did not discard the species erroneously. For ten species (noted with an asterisk in Additional file 1), the presence of significant morphological variation led us to create a second entity of these species called a *variation.*

**Figure 1:**
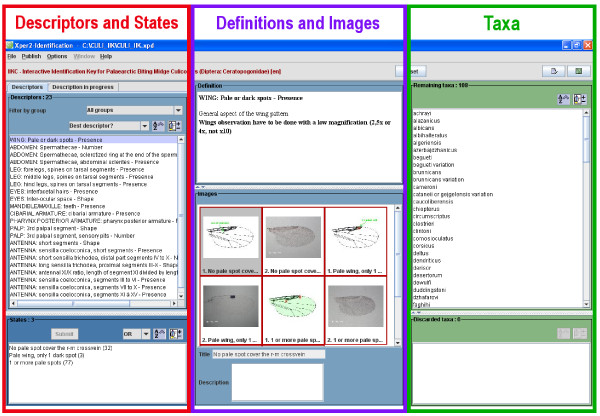
**Initial screen of IIKC upon opening program.** On the left side, the descriptor list and their states (red rectangle); on the right side the remaining and discarded taxa (green rectangle) and, in the middle definitions and pictures (violet rectangle) of either descriptors, states or taxa according to the selection.

Morphological characters were image-captured using a Zeiss® microscope equipped with a Motic® camera, and were processed with the Gimp© editor version 2.6.2, (Free Software Foundation, Boston, USA). The list of morphological characters (Table 1) and state of characters were chosen through discussion with international experts at a meeting on *Culicoides* taxonomy in Strasbourg in 2009 (http://medreonet.cirad.fr/news/2009_taxonomy). A total of 73 taxa were characterised with 434 images (5.9 pictures/taxon) and 71 additional diagrams were also produced. Six rare taxa were not illustrated because of the poor quality of the specimens available. Among the 61 descriptors used, 60 were morphological characters (27 wing, 14 abdominal, 16 head and 3 leg characters) and one referred to the known geographical distribution (Table 1). The geographical descriptor was based on publications and included the 16 countries gathered around a European project (http://medreonet.cirad.fr/): Algeria, Belgium, Denmark, France, Germany, Greece, Italy, Morocco, Netherlands, Portugal, Spain, Sweden, Switzerland, Tunisia, Turkey and the United Kingdom. The graphical user interface is illustrated as a screenshot (Figure 1). The middle section of the interface was dedicated to definitions and images of both descriptors (on the left part) and taxa (on the right part). As a quick start guide, notices on “How to install” and “How to identify” were added.

**Table 1:** Descriptors and descriptor codes used for IIKC

**Descriptors**	**code**
WING: Pale or dark spots - Presence	W01
WING: 2nd rad cell, covered by pale spot, costal-tip part	W02
WING: r5 and m1, pale spots, distal part - Presence	W03
WING: r5 and m1, pale spots, distal part - Size	W04
WING: r5 and m1, pale spots, distal part - Connection	W05
WING: r5 and m1, pale spots distal part - Position	W06
WING: m2, pale spot, distal part - Presence	W07
WING: m1, pale spot, from proximal to median part - Presence	W08
WING: m1, pale spot layer and cross the veins M1 and M2 - Presence	W09
WING: m2, pale spot/area, from proximal to median part - Presence	W10
WING: m2, pale spot over r-m cross vein fused with the m2 spot which layers and crosses vein M2 - Presence	W11
WING: m, pale spot/area - Presence	W12
WING: r5, 4th pale costal spot (p.c.s.) versus 3rd dark costal spot (d.c.s.) - Size	W13
WING: r5, area of 4th p.c.s. bigger than 3rd d.c.s. - Shape of the 3rd d.c.s.	W14
WING: anal cell, pale spot in distal part - Presence	W15
WING: m4, center spot - Presence and Colour	W16
WING: r-m crossvein, dark spot in the corner with M1 vein- Presence	W17
WING: arculus, dark spot under arculus - Presence	W18
WING: M1, pale spot/band spanning the vein - Presence	W19
WING: M1, pale spot in the median part - Position	W20
WING: M2, pale spot/band spanning the vein - Presence	W21
WING: M1, M2 and M3 + 4, at least 1 pale spot/area, abuts wing margin, apex of veins in distal part - Presence	W22
WING: M1, M2 and M3 + 4, pale spots surrounded by dark area, apex of veins - Shape	W23
WING: M2, dark spot in proximal part - Shape	W24
WING: Pale wing with only 2 dark areas on Cu1 and 2nd rad cell - Presence	W25
WING: m and anal cells, macrotrichia abundance - Presence	W26
WING: anal cell, dark area abuts wing margin - Presence	W27
ABDOMEN: Spermathecae - Number	A01
ABDOMEN: Spermathecae, sclerotized ring at the end of the spermathecal duct - Presence	A02
ABDOMEN: Spermathecae, sclerotized ring at the end of the spermathecal duct - Shape	A03
ABDOMEN: 1 or 2 spermathecae, pigmented neck - Presence	A04
ABDOMEN: 1 Spermatheca - Shape	A05
ABDOMEN: 1 spermatheca, curved shape - Presence	A06
ABDOMEN: 1 spermatheca, spermathecal duct swollen - Presence	A07
ABDOMEN: 1 spermatheca, spermathecal duct - Length	A08
ABDOMEN: 2 spermathecae - Shape	A09
ABDOMEN: Spermathecae, abdominal sclerites - Presence	A10
ABDOMEN: Spermathecae, abdominal sclerites - Shape	A11
ABDOMEN: 2 spermathecae - Size	A12
ABDOMEN: 3 spermathecae - Shape	A13
ABDOMEN: 3 spermathecae - Texture	A14
EYES: interfacetal hairs - Presence	H01
EYES: Inter-ocular space - Shape	H02
MANDIBLE/MAXILLE: teeth - Presence	H03
CIBARIAL ARMATURE: cibarial armature - Presence	H04
PHARYNX POSTERIOR ARMATURE: pharynx posterior armature - Presence	H05
PALPUS: 3rd palpal segment - Shape	H06
PALPUS: 3rd palpal segment, sensory pits - Number	H07
PALPUS: 3rd palpal segment, single sensory pit - Opening versus depth	H08
ANTENNA: short segments - Shape	H09
ANTENNA: sensilla coeloconica, short segments - Presence	H10
ANTENNA: short sensilla trichodea, distal part segments IV to X - Number	H11
ANTENNA: long sensilla trichodea, proximal segments III-X - Shape	H12
ANTENNA: antennal XI/X ratio, length of segment XI divided by length of segment X - Range	H13
ANTENNA: sensilla coeloconica, segments III to VI - Presence	H14
ANTENNA: sensilla coeloconica, segments VII to X - Presence	H15
ANTENNA: sensilla coeloconica, segments XI à XV - Presence	H16
LEG: forelegs, spines on tarsal segments - Presence	L01
LEG: middle legs, spines on tarsal segments - Presence	L02
LEG: hind legs, spines on tarsal segments - Presence	L03
GEOGRAPHICAL	G01

Concerning wing descriptors, the lower-case *r* and *m* referred to respectively radial and median cells and the upper-case *M* and *Cu* to the median and cubital nervures.

### Database management system

Xper^2^ version 2.0 [15] was selected to edit and to manage the morphological database and to create the interactive key. It does not require advanced programming and can be freely downloaded at http://lis-upmc.snv.jussieu.fr/lis/?q=ressources/logiciels/xper2. Xper² is a versatile software for editing, managing, storing and providing for on-line publishing of taxonomic knowledge. Several tools are available in order to facilitate the daily work of its users: the checkbase function prevents inconsistencies, the summary function can provide an overview of the whole knowledge base and items are easily compared within a matrix. In addition, Xper^2^ allows the use of operators to take into account the treatment of polymorphism or uncertainty. The descriptors can be sorted according to their discriminant power using three indexes: one is unique to the software, Xper² original sort, and two are well-known mathematical indexes, the Sokal and Michener sort, and the Jaccard sort.

IIKC was validated by 6 users with different levels of expertise in *Culicoides* identification. Two were *beginners* on *Culicoides* taxonomy, defined as possessing little experience with identification keys in general (users 1 and 2); two were defined as of *intermediate* skill, with experience with mosquitoes and tick taxonomy, but none with *Culicoides* (user 3 and 4); and two were defined as *advanced* users with expertise on *Culicoides* taxonomy and identification keys (user 5 and 6). A total of 37 slide-mounted female *Culicoides* representing 34 species morphologically confirmed by two experts, were sent without identifying labels to users. Specimens were recorded with a reference number and the trapping location.

A questionnaire was sent to the users to record the final species identifications, the time required for identification and the level of confidence the user attached to each identification. To begin the identification process, users activated the *Xper original sort* and then freely selected the descriptors among the list sorted in a decreasing order of discriminant power, *i.e.* from the descriptors that will best discriminate the taxa to the least. Identification slide orders were randomly selected for each user. After each specimen identification, users saved the identification pathway history (automatically generated by the Xper^2^ software) with the state of characters selected. To avoid heterogeneity in identification effort, users were recommended to complete only one identification process per specimen. Each original step was checked afterwards to see whether each morphological state chosen by the user discarded the correct taxon or not. The selection of a morphological state was considered as an *error* if the correct taxon was discarded and as a *success* if not. Each morphological selection of the step *n* was checked independently of the results of the step *n-1* meaning a success due to a good morphological observation could be possible at the step *n* even if an error occurred at *n-1* discarding the correct taxon. A step was considered as an observation from which *success* and *error* were computed, if the step discarded at least one taxon. Each of the 222 identification pathway histories generated by the six users was then checked to compute the quality of user observations. An observation (step) was computed as *error* if the selected state discarded the correct taxa and as *success* when the correct taxa remained in the taxa list*.*

### Analysis

Data from the validation step was analyzed with a factorial component analysis using the *ade4* package of R software [16]. The statistical tests were computed with R software. Differences of success frequency between users and between the user classes were investigated by a chi-squared test. Normality of dataset and subsets were assessed with the Shapiro-Wilks test. The mean differences of non-normal data were explored using the Kruskal-Wallis test. In case of significance of the latest, *kruskalmc* function of the *pgirmess* R package and the Wilcoxon test were used to investigate multiple comparisons between classes and within two classes.

## Results

### Database contents and structure

**Figure 2:**
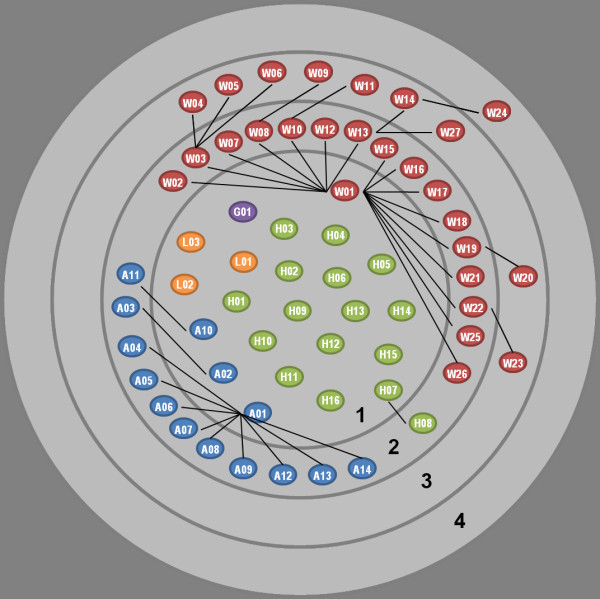
**IIKC database structure.** The four hierarchical levels are represented by grey circles and are numbered 1 to 4 (black). The first level gathered the 23 descriptors available at the start of identification. Descriptor logical dependency between two descriptors was shown by a black line meaning a particular state of the descriptor into level *n* have to be selected to “unlock” the one into the level *n + 1.* The unlocked descriptors were incremented in the list of the descriptors available to user.

IIKC database structure was based on descriptor dependency, with four hierarchical levels (Figure 2). All identifications started with a choice of 23 descriptors (level 1), 28 on level 2, 9 on level 3 and only 1 on level 4. Descriptors for level 1 are not inter-related meaning that selection between each of them is possible (Figure 2). Logical dependencies then determine the availability pathway of descriptors between levels 2-4 by removing redundant descriptors following the selection of particular characters.

**Figure 3:**
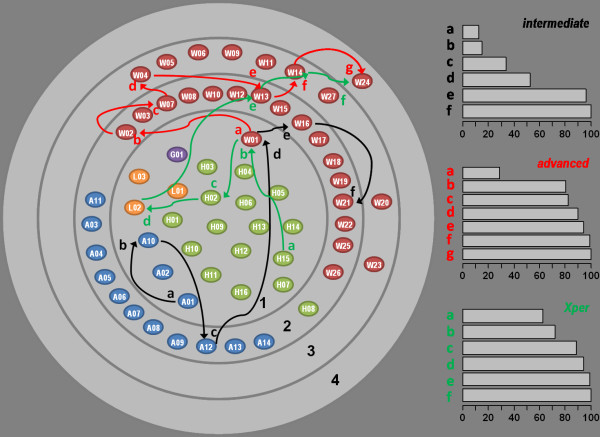
**Examples of end user pathways,*intermediate*and*advanced*user, for the identification of*C. newsteadi*, and the pathway following the xper sort.** Histogram showed the percentage of discarded taxa at each step for each pathway. Letters *a* to *f* or *g* corresponded to the identification step 1 to 6 or 7 on the scheme and on the histogram; the black, green and red letters/arrows corresponded to respectively *intermediate*, *advanced* user and the *xper* sort.

As expected, identification pathways vary according to the user of the programme. As an illustration of this, a comparison of the selection process by two users to identify correctly *C. newsteadi* was documented (Figure 3) and compared with the optimised pathway following the “Xper original sort”. The number of steps, characters used and the final descriptors allowing discrimination of *C. newsteadi* were different. Comparing the first step of these three pathways, the optimised one discarded 62% of taxa compared to respectively 12% and 28% for the intermediate and advanced users.

### Analysis of IIKC validation

A factorial component analysis between the different variables (slide order, identification time, confidence percentage and number of descriptors) was performed (data not shown). Projections of either slides or users to the factorial axis did not reveal any pattern. Identification data (identification time, success or failure to identify correctly the specimen, number of descriptors used) for each user was then individually analysed.

Successful identification took a median of seven steps (inter-quartile range of 3) and seven minutes (inter-quartile range of 5). Identification success rate varied according to the species concerned (Table 2). Success rates differed significantly between users (chi-squared test, p = 0.0033) and between levels of experience (chi-squared test, p = 0.0011) and ranged from 35.1% to 81.1%. By level of experience, the beginners successfully identified 44.6% of slides, the intermediates 56.8% and the advanced 74.3%. Each specimen, however, was correctly identified at least once within the group and four specimens, (*C. nubeculosus*, *C. parroti*, *C. saevus* and *C. semimaculatus*) were correctly identified by all users (Table 2). For all three user categories, median confidence was 60% for failed identifications and 80% for successfully identified specimens.

**Table 2:** For each species used for the validation, number of successful identifications, number of descriptors used by end user when the identification was correct, and theoretical number of descriptors following strictly the list of the Xper original sort

**Species**	**Nb of Success**	**Users’ step**				**Nb Xper step**
		**Min**	**Mean**	**Max**	**Sd**	
*C. begueti*	3	8	9.3	10	0.9	7*
*C. brunnicans*	3	3	6	9	2.4	6
*C. cameroni*	3	3	6.3	9	2.5	5*
*C. chiopterus*	3	4	6.3	10	2.6	7
*C. circumscriptus*	4	5	6	8	1.2	5*
*C. dewulfi*	4	9	9.7	11	0.8	10
*C. fascipennis*	2	9	9.5	10	0.5	8*
*C. fascipennis*	1	9	9	9	0	8*
*C. festivipennis*	4	5	7	10	1.9	6*
*C. haranti*	3	6	7.3	9	1.2	7*
*C. heliophilus*	3	7	8	10	1.4	7*
*C. imicola*	5	4	5.8	8	1.5	6*
*C. kibunensis*	1	11	11	11	0	9*
*C. longipennis*	3	5	8.7	11	2.6	7*
*C. lupicaris*	2	6	7.5	9	1.5	7*
*C. minutissimus*	3	3	4	5	0.8	5
*C. montanus*	5	5	6.8	9	1.3	9
*C. newsteadi*	4	6	8.5	14	3.2	7*
*C. nubeculosus*	6	3	3.7	5	0.7	5
Obsoletus complex	5	9	9.4	11	0.8	10
Obsoletus complex	3	7	8.3	10	1.2	10
*C. paradisionensis*	1	8	8	8	0	7*
*C. parroti*	6	3	4.2	5	0.9	5
*C. picturatus*	1	7	7	7	0	6*
*C. picturatus*	3	7	8.7	10	1.2	7*
*C. poperinghensis*	1	8	8	8	0	7*
*C. pulicaris*	5	6	7	8	0.9	7*
*C. punctatus*	5	6	8.4	15	3.3	7*
*C. riebi*	1	9	9	9	0	8*
*C. riethi*	3	6	7	8	0.8	6*
*C. riouxi*	5	4	4.8	5	0.4	6
*C. saevus*	6	2	3	4	0.8	4
*C. segnis*	5	3	6.2	8	1.9	6*
*C. sejfadinei*	5	3	3	3	0	6
*C. semimaculatus*	6	3	7	8	1.8	6*
*C. stigma*	3	4	4.3	5	0.5	4*
*C. vexans*	3	4	7	11	2.9	5*

* mentioned the 25 out of the 37 specimens (68%) would have been correctly identified quicker than following the user’s choices.

For users, successful identifications were achieved in an average of 6.6 steps, with a minimum of two steps (for *C. saevus*) and a maximum of 15 steps (for *C. punctatus*). No significant difference was observed between the number of descriptors used when identification failed, succeeded or both, either between users or user’s class (Kruskal-Wallis test, p > 0.05). For all users, the identification time was significantly higher when identification failed than when identification succeeded (one-sided Wilcoxon test, p = 0.0093). No significant differences were noted, however, either between users or within the user’s class (all Kruskal-Wallis tests, p > 0.05) although complete data was not available for the beginner class.

**Figure 4:**
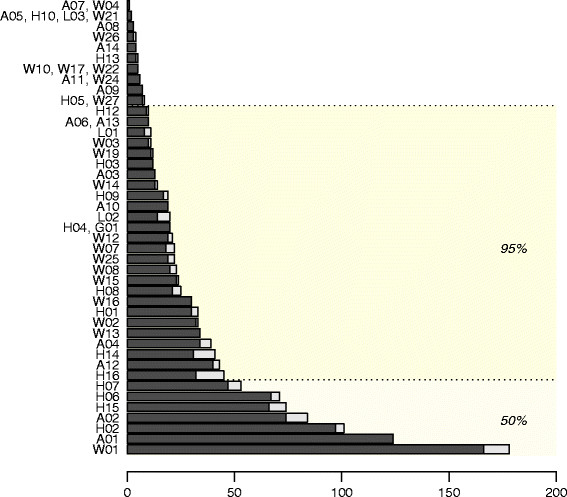
**Numbers of observations for each descriptor used (n = 1,397).** Black bars represent successful observations and grey ones those which failed. The light area gathered the seven descriptors, which represents 50% of total observations and the pigmented area 95% of the whole. Bars were ordered from the above to the top by total decreasing. The stars were added for the descriptors leading to error superior to 10%.

**Figure 5:**
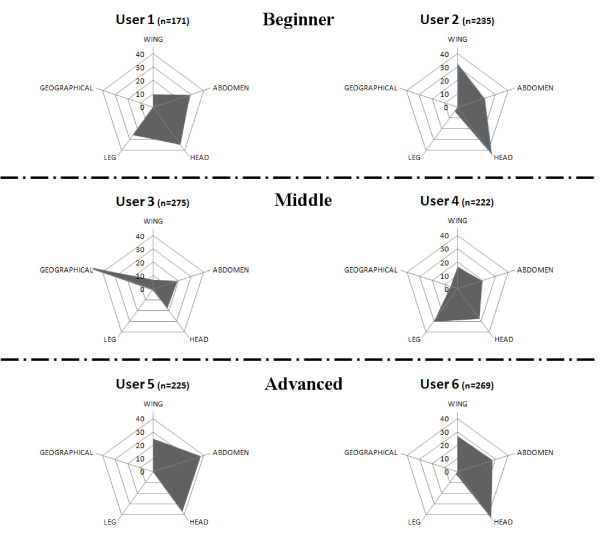
**Descriptor groups used by the different users (in percentage) with** ***n*** **as the total number of observations made by each user.**

Eight out of 61 descriptors were not used during the validation (wing characters: W05, W06, W09, W11, W18, W20, W23 and head character: H11). Users selected a total of 1,397 character states for 53 descriptors of which seven descriptors represented 50% of the descriptors used, namely by decreasing order: W01, A01, H02, A02, H15, H06 and H07 (Figure 4). Most successful descriptors included the use of A01 and H02, which led to error in less than 5% of cases and W01 and H06, which led to error in less than 10%. Similarly, the sclerotized ring (A02), sensilla distribution (H15) and sensory pits (H07) were used with 12, 11 and 11% of error respectively. In all, 36 descriptors represented 95% of use of the key. Each user demonstrated a particular pattern of preference for use of descriptor groups (Figure 5). As an example only one user made an initial sort according to the origin of the specimen. The beginners and the intermediate users also utilised very different patterns of descriptor use. The advanced group had a more similar pattern giving priority to observation of the head followed by the abdomen and the wings and additionally avoided observing legs and using the geographical descriptor.

## Discussion

This study has produced and validated IIKC, the first open-access electronic key for *Culicoides* to be developed worldwide and has demonstrated some of the advantages and disadvantages in providing taxonomic information to a range of different users using e-tools. The validation was carried out with the aim of investigating the impact that the key could have upon users ranging from beginners with no experience of either electronic keys or biting midge identification, to those carrying out *Culicoides* identification daily, but whom were trained in a different laboratory and country (in this case the United Kingdom). This was used to reflect the scenario that in the event of the incursion of a *Culicoides*-borne pathogen, staff with highly diverse levels of experience are expected to contribute to identification of *Culicoides* across a wide geographical range. In addition, rather than just including *Culicoides* species that were expected, *a priori*, to act as vectors, the validation included a challenging and realistically wide range of specimens that might be collected at light in the region (allowing a fuller understanding of species diversity) [9,17-20].

The validation results provided valuable information regarding the likely accuracy of surveys conducted by users of different levels of expertise and also highlighted improvements that could be made to IIKC, allowing an assessment of the degree to which specialist coaching would still be required in an outbreak situation. The relatively low success of the advanced users (74.3%) could be explained by two phenomena. First, we cannot underestimate the fact that all users may be puzzled when they discovered the key for the first time during the trial. Indeed, independently of the difficulty of species identification, the random order of specimens during the identification process demonstrated that half of the errors occurred for the first 14 specimens. This observation was confirmed by user feedback, which estimated that around 10 identification processes were necessary to feel comfortable with the software interface. The absence of errors occurring for the last seven specimens would indicate a tendency to reach 100% success rate for the advanced users. Secondly, the advanced users have realized afterwards that their observations of the subjective characters, sometimes did not match with their final and confirmed diagnosis. Their observations of the non-corresponding subjective characters were computed as errors in this analysis, and consequently downgraded their success rates. Such subjective characters e.g. the neck of the spermathecae or the shape of palpus, have revealed a need to update data eventually by coding them as polymorphic. Some species are clearly easier to identify, even for beginners, due to distinctive features, which are simple to observe, such as the swollen duct of the unique spermatheca of *C. nubeculosus.* Apart from such unique features, the species with wings that have well marked patterns were less problematic for users. On the contrary, the species causing the most difficulty were those with only two small and faint spots on the wings such as *C. paradisionensis*. Without experience regarding the intraspecific range of variations on *Culicoides* wing patterns, the difficulty in determining such a wing as with or without spots is real. To prevent errors due to subjective state of characters, database updates would be focused on making them clearer. Similarly, in case of a doubt in choosing the right character, we will enhance the functionality of Xper to allow users selecting more than one state of character. Primarily focused on microscopic characters, other features such as the coloration of the dorsum of the thorax e.g. useful for *C. flavipulicaris* or *C. clastrieri*, the scutum pattern *e.g. C. nubeculosus* or *C. riethi*, observable on specimens in alcohol would be added in the future to allow users to make a first sort before confirmation by slide-mounting.

The number and quality of images available in the IIKC guides users through the identification process, allowing them to assess their confidence in the result produced. Its flexibility through the use of a multi-entry system is also demonstrated by the fact that different users can use two different pathways to identify *C. newsteadi*, depending upon the characters they feel confident in applying (Figure 5). This system also has an additional advantage in allowing avoidance of descriptors that correspond to a damaged/missed anatomical part in the specimen. With experience, the user behaviour seems to concentrate upon characters of the head and the abdomen more than on the wings and very few are observed on the legs. Advanced users additionally never used the geographical character, probably concluding through their experience that most species are widely distributed.

Beyond the 36 descriptors that represented 95% of the whole observations, 8 descriptors were never used for several reasons. The distribution of the short sensilla trichodea (H11) was probably not used because of the difficulty to observe them without experience. Additionally, characters W05 and W11 were special features specific to *C. caucoliberensis* and *C. simulator* respectively, which were absent from the validation trial. The other five descriptors - W06, W09, W18, W20 and W23 – were not special features discriminant of species. In these cases the position on the list could have been a determining factor in their use. At present it is not possible to add weights to the descriptors either in terms of ease of use or specificity, however, this is planned in forthcoming developments and will take into account the feedback of those involved in the trial.

Technically, the software itself is relatively straightforward to operate and assists accurate identification in several ways. Uptake of the various tools provided within the programme is of interest in approving the acceptability to different user groups. To assist in identification, the software allows three options: Option 1 allows managing uncertainty by using logical operators (like AND, XOR, NOT) to select several choices within the key. Even though this could be useful on occasion for difficult or subjective characters (like sensilla distribution or the palpus shape), none of the users used this function during the validation although this may be through a lack of awareness or confidence. The second option is to define a mismatch threshold when performing identifications. Each value for this option was not evaluated and no recommendation could be made. Nevertheless, an observed effect to increase the mismatch threshold is to increase the number of steps to identify. This is balanced by the fact that the validation protocol revealed that identifications requiring a lot of steps often lead to a higher number of errors. The last option assisting in identification is to compare the selected taxa by producing a matrix summarizing descriptions, with an easy to read colour-code indicating whether a character is discriminating, partially discriminating or not discriminating. This latter option could be used to improve the user knowledge and his confidence by checking which characters are discriminating among the selected taxa.

To date, all available identification tools for *Culicoides* are based upon single-access keys and are in specialist journals or PhD theses, which are often not easily available to new users. IIKC sits between very general databases that act as a repository for a wide variety of information concerning *Culicoides* biology (e.g. http://www.culicoides.net or http://bluetongue.cirad.fr/) and published keys, and will allow at least basic competence to be developed by users. While the identifications made by beginners will still require secondary confirmation by experts (and these confirmations in themselves are prone to subjective biases), the provision of the key online and with access to other workers will significantly improve the consistency of *Culicoides* identification in Northern Europe. Taxonomy as a discipline has advanced substantially through internet-based resources as it is reliant upon both detailed description and high quality images [21]. While not replacing the specialist’s role in training new taxonomists, IIKC provides a complementary mechanism as a back-up tool for experts. According to the trial results, IIKC will be improved by weighting descriptors that are easy to observe, by evaluating the mismatch thresholds for beginners, by developing definitions and images for difficult descriptors and by adding illustrations and information of particular features of species. A scientific committee will be organised to validate updates, to discuss new species or synonymies and to evaluate new systematic or taxonomic changes.

**IIKC is available in a cd-rom format upon request from the authors or can be downloaded from the following website**http://www.iikculicoides.net.

## Conclusion

IIKC, an Interactive Identification Key for females of the species of *Culicoides* of the West Palaearctic region, is a multi-entry key providing taxonomic information for 98 species and 10 variants with 837 photographic images and illustrations. In addition to the key, users can browse the database including morphological data for 60 characters, synonymies and geographical distribution among 14 countries. Validated by six users with a various range of experience, IIKC appears to be straightforward to use. In addition to the key, the huge amount of taxonomic information available acts a back-up source for the e-taxonomy of the genus *Culicoides.* The development and the free sharing between beginners and experts of the e-taxonomy such as IIKC for *Culicoides* and more generally for arthropods involved in pathogen transmission will unlock the taxonomic knowledge to identify species and therefore will give better insights into the ecology and dynamics of these groups, helping to standardise vector surveillance strategies across countries.

## Competing interests

The authors declare that they have no competing interests.

## Authors’ contributions

TB, BM & JCD initiated the study; BM & DC drafted the database and RVL & VU contributed to the final database structure; BM & JCD collected the morphological data; JCD made the drawings; BM took the pictures and designed the scheme to define characters; SC, MLSR, EC, TB & CG organized the trial in their laboratory; TB & BM carried out the data analysis and interpretation; BM, CG, CCS & SC wrote the manuscript; All authors read and approved the final version of the manuscript.

## Supplementary Material

Additional file 1 List of the 98 species represented in IIKC. Descriptor names, year of description and subgeneric affiliation are given following Borkent [22] except for *C. dendriticus, C. lupicaris, C. remmi C. submaritimus* which are here treated as valid species. Click here for file
