# Supplementary material for: Development and validation of IIKC: an interactive identification key for Culicoides (Diptera: Ceratopogonidae) females from the Western Palaearctic region
Source: Parasit Vectors. 2012 Jul 9;5:137. doi: 10.1186/1756-3305-5-137 (PMC3483010; doi:10.1186/1756-3305-5-137)
Supplement: Additional file 1 — List of the 98 species represented in IIKC. Descriptor names, year of description and subgeneric affiliation are given following Borkent [22] except for C. dendriticus, C. lupicaris, C. remmi C. submaritimus which are here treated as valid species. [file 1756-3305-5-137-S1.doc]

**Additional file 1.**

List of the 98 species represented in IIKC. Descriptor names, year of description and subgeneric affiliation are given following Borkent [22] except for *C. dendriticus*, *C. lupicaris*, *C. remmi* and *C. submaritimus* which are here treated as valid species.

The species marked with an asterisk are divided in two taxonomic entities: a typical form and a morphological variant form. Morphological data of the species annotated with *c* were observed from types in the Callot and Kremer collection (Strasbourg, France) and *d* for from a type in the Delécolle collection (Strasbourg, France). Data for all others species were studied from specimens in the collection of the Institut de Parasitologie et de Pathologie Tropicale de Strasbourg, France. Specimens were identified by J.C. Delécolle. Countries of origin of the specimens were abbreviated as follow: CH, Switzerland; DZ, Algeria; FR, France; IT, Italia; KZ, Kazakhstan; MA, Morocco; TN, Tunisia.

| Species | Type | Country |
| --- | --- | --- |
| *C. (Avaritia) chiopterus* (Meigen), 1830 | *-* | FR |
| *C. (Avaritia) dewulfi* Goetghebuer, 1936 | *-* | FR |
| *C. (Avaritia) imicola* Kieffer, 1913 | *-* | FR |
| *C. (Avaritia) montanus* Shakirzjanova, 1962 | *-* | FR |
| *C. (Avaritia) obsoletus* (Meigen), 1818 / *C. (A.) scoticus* Downes and Kettle, 1952 | *-* | FR |
| *C. (Beltranmyia) circumscriptus* Kieffer, 1918 | *-* | FR |
| *C. (Beltranmyia) desertorum* Gutsevich, 1959 | *-* | KZ |
| *C. (Beltranmyia) salinarius* Kieffer, 1914 | *-* | FR |
| *C. (Beltranmyia) sphagnumensis* Williams, 1955 | *c* | FR |
| *C. (Culicoides) deltus* Edwards, 1939 | *-* | FR |
| *C. (Culicoides) fagineus* Edwards, 1939 | *-* | FR |
| *C. (Culicoides) flavipulicaris* Dzhafarov, 1964 | *-* | FR |
| *C. (Culicoides) grisescens* Edwards, 1939 | *-* | FR |
| *C. (Culicoides) impunctatus* Goetghebuer, 1920 | *-* | FR |
| *C. (Culicoides) lupicaris* Downes and Kettle, 1952 | *-* | FR |
| *C. (Culicoides) newsteadi* Austen, 1921 | *-* | FR |
| *C. (Culicoides) pulicaris* (Linnaeus), 1758 | *-* | FR |
| *C. (Culicoides) punctatus* (Meigen), 1804 | *-* | FR |
| *C. (Culicoides) remmi* Damian-Georgescu, 1972 | *-* | IT |
| *C. (Culicoides) subfagineus* Delécolle and Ortega, 1998 | *-* | FR |
| *C. (Monoculicoides) nubeculosus* (Meigen), 1830 | *-* | FR |
| *C. (Monoculicoides) parroti* Kieffer, 1922 | *-* | FR |
| *C. (Monoculicoides) puncticollis* (Becker), 1903 | *-* | FR |
| *C. (Monoculicoides) riethi* Kieffer, 1914 | *-* | FR |
| *C. (Monoculicoides) stigma* (Meigen), 1818 | *-* | FR |
| *C. (Oecacta) kingi* Austen, 1912 | *-* | DZ |
| *C. (Pontoculicoides) saevus* Kieffer, 1922 | *-* | DZ |
| *C. (Pontoculicoides) sejfadinei* Dzhafarov, 1958 | *-* | DZ |
| *C. (Pontoculicoides) tauricus* Gutsevich, 1959 | *-* | FR |
| *C. (Silvaticulicoides) achrayi* Kettle and Lawson, 1955 | *-* | FR |
| *C. (Silvaticulicoides) fascipennis* (Staeger), 1839 | *-* | FR |
| *C. (Silvaticulicoides) pallidicornis* Kieffer, 1919 | *-* | FR |
| *C. (Silvaticulicoides) picturatus* Kremer and Deduit, 1961 | *-* | FR |
| *C. (Silvaticulicoides) subfasciipennis* Kieffer, 1919 | *** | FR |
| *C. (Synhelea) corsicus* Kremer, Leberre and Beaucournu-S., 1971 | *c* | FR |
| *C. (Synhelea) marcleti* Callot, Kremer and Basset, 1968 | *-* | DZ |
| *C. (Wirthomyia) minutissimus* (Zetterstedt), 1855 | *-* | FR |
| *C. (Wirthomyia) reconditus* Campbell and Pelham-Clinton, 1960 | *-* | FR |
| *C. (Wirthomyia) riouxi* Callot and Kremer, 1961 | *-* | FR |
| *C. (Wirthomyia) segnis* Campbell and Pelham-Clinton, 1960 | *-* | FR |
| *C. alazanicus* Dzhafarov, 1961 | *c* | FR |
| *C. albicans* (Winnertz), 1852 | *-* | FR |
| *C. albihalteratus* Goetghebuer, 1935 | *c* | FR |
| *C. algeriensis* Clastrier, 1957 | *-* | DZ |
| *C. azerbajdzhanicus* Dzhafarov, 1962 | *-* | DZ |
| *C. begueti* Clastrier, 1957 | *** | FR |
| *C. brunnicans* Edwards, 1939 | *** | FR |
| *C. cameroni* Campbell and Pelham-Clinton, 1960 | *-* | FR |
| *C. cataneii* Clastrier, 1957 | *-* | FR |
| *C. caucoliberensis* Callot, Kremer, Rioux and Descous, 1967 | *c* | FR |
| *C. clastrier* Callot, Kremer and Deduit, 1962 | *c* | FR |
| *C. clintoni* Boorman, 1984 | *-* | FR |
| *C. comosioculatus* Tokunaga, 1956 | *-* | CH |
| *C. dendriticus* Boorman, 1976 | *-* | FR |
| *C. derisor* Callot and Kremer, 1965 | *c* | FR |
| *C. duddingstoni* Kettle and Lawson, 1955 | *-* | FR |
| *C. dzhafarovi* Remm, 1967 | *c* | FR |
| *C. faghihi* Navai, 1971 | *-* | DZ |
| *C. festivipennis* Kieffer, 1914 | *** | FR |
| *C. furcillatus* Callot, Kremer and Paradis, 1962 | *c* | FR |
| *C. gejgelensis* Dzhafarov, 1964 | *-* | FR |
| *C. griseidorsum* Kieffer, 1918 | *-* | FR |
| *C. haranti* Rioux, Descous and Pech, 1959 | *** | FR |
| *C. heliophilus* Edwards, 1921 | *-* | FR |
| *C. helveticus* Callot, Kremer and Deduit, 1962 | *c* | CH |
| *C. heteroclitus* Kremer and Callot, 1965 | *c* | FR |
| *C. indistinctus* Khalaf, 1961 | *-* | FR |
| *C. jumineri* Callot and Kremer, 1969 | **c* | TN |
| *C. jurensis* Callot, Kremer and Deduit, 1962 | *c* | FR |
| *C. kibunensis* Tokunaga, 1937 | *-* | FR |
| *C. kurensis* Dzhafarov, 1960 | *-* | FR |
| *C. landauae* Kremer, Rebholtz-Hirtzel and Bailly-Choumara, 1975 | *-* | MA |
| *C. langeroni* Kieffer, 1921 | *-* | DZ |
| *C. longipennis* Khalaf, 1957 | *-* | FR |
| *C. maritimus paucisensillatus* Callot, Kremer and Rioux, 1963 | **c* | FR |
| *C. malevillei* Kremer and Coluzzi, 1971 | *c* | FR |
| *C. maritimus* Kieffer, 1924 | *** | FR |
| *C. odiatus* Austen, 1921 | *** | FR |
| *C. pallidus* Khalaf, 1957 | *-* | DZ |
| *C. paolae* Boorman, 1996 | *-* | FR |
| *C. paradisionensis* Boorman, 1988 | *d* | FR |
| *C. pictipennis* (Staeger), 1839 | *-* | FR |
| *C. poperinghensis* Goetghebuer, 1953 | *-* | FR |
| *C. pseudolangeroni* Kremer, Chaker and Delecolle, 1981 | *-* | DZ |
| *C. pseudopallidus* Khalaf, 1961 | *-* | DZ |
| *C. ravus* de Meillon, 1936 | *-* | DZ |
| *C. riebi* Delécolle, Mathieu and Baldet, 2005 | *-* | FR |
| *C. sahariensis* Kieffer, 1923 | *c* | FR |
| *C. santonicus* Callot, Kremer, Rault and Bach, 1966 | **c* | FR |
| *C. semimaculatus* Clastrier, 1958 | *-* | FR |
| *C. sergenti* Kieffer, 1921 | *-* | DZ |
| *C. shaklawensis* Khalaf, 1957 | *-* | FR |
| *C. simulator* Edwards, 1939 | *-* | FR |
| *C. submaritimus* Dzhafarov, 1962 | *-* | FR |
| *C. truncorum* Edwards, 1939 | *c* | FR |
| *C. univittatus* Vimmer, 1932 | *c* | FR |
| *C. vexans* (Staeger), 1839 | *-* | FR |
| *C. vidourlensis* Callot, Kremer, Molet and Bach, 1968 | *c* | FR |
